# Vascular dysfunction and body mass index in African adults with HIV

**DOI:** 10.1186/s12872-023-03093-2

**Published:** 2023-02-03

**Authors:** Longa Kaluba, Theresa Chikopela, Fastone Goma, Mordecai Malambo, Wilbroad Mutale, Douglas C. Heimburger, John R. Koethe

**Affiliations:** 1 School of Medicine, Cavendish University Zambia, Lusaka, Zambia; 2 Department of Human Physiology, Faculty of Medicine, Lusaka Apex University, Lusaka, Zambia; 3 Department of Physiological Sciences, School of Medicine, University of Zambia, Lusaka, Zambia; 4 Department of Health Policy and Management, School of Public Health, University of Zambia, Lusaka, Zambia; 5 Vanderbilt Institute for Global Health and Department of Medicine, Vanderbilt University Medical Centre, Nashville, TN USA; 6 Department of Internal Medicine, School of Medicine, University of Zambia, Lusaka, Zambia

**Keywords:** Arterial stiffness, Augmentation Index, HIV, Body mass index, Sub-Saharan Africa

## Abstract

**Background:**

Impaired vascular compliance is common among persons with HIV (PWH) and a risk factor for cardiovascular disease (CVD), though many studies documenting this are from regions with a high prevalence of overweight and obesity. The prevalence and characteristics of impaired vascular compliance among PWH with low body mass index (BMI) is not well described, particularly in sub-Saharan Africa (SSA) where the majority of PWH live, a low BMI is more common, and the burden of CVD is rising.

**Aim:**

To assess non-invasive vascular compliance measurements, including augmentation index (AIX), pulse wave velocity (PWV) and pulse waveforms, in underweight, normal weight, and overweight PWH on long-term antiretroviral therapy (ART) in SSA.

**Methods:**

A cross-sectional study among PWH on ART at the University Teaching Hospital in Lusaka, Zambia. All participants had been on a regimen of efavirenz, emtricitabine, and tenofovir disoproxil fumarate for five or more years. Carotid-femoral PWV (cfPWV), carotid-radial PWV (crPWV), and the corresponding augmentation indexes (cfAIX and crAIX), were measured in all participants, in addition to aortic pressure waveforms, classified as type A, B, C and D according to reflected wave timings and amplitude. Multiple linear regression assessed relationships between demographic and clinical factors with vascular measurement endpoints.

**Results:**

Ninety one PWH on long-term ART were enrolled; 38 (42%) were underweight (BMI < 18.5 kg/m^2^), 43 (47%) were normal weight (18.5–24.9 kg/m^2^) and 10 (11%) were overweight (> 25 kg/m^2^). Median age was 41, 40 and 40 years, among the three groups, respectively, and the proportion of women increased with BMI level. Overweight participants had a 39% higher cfAIX compared to normal-weight participants, while being underweight was associated with 27% lower cfAIX, after adjusting for age, sex and blood pressure (*P* = 0.02 and *P* = 0.01, respectively), but measurements of cfPWV, crPWV and crAIX did not differ.

**Conclusion:**

Underweight PWH in SSA had lower cfAIX measurements compared to normal weight individuals, indicating less arterial stiffness. However, similar cfPWV, crPWV and crAIX values among the underweight and overweight PWH suggest a low BMI may not confer substantial protection against impaired vascular compliance as a contributor to CVD risk among individuals on ART.

## Introduction

Globally, the prevalence of cardiovascular diseases (CVD) has tripled over the past two decades [1]. The risk of cardiovascular events, namely myocardial infarction, heart failure and stroke, among persons with HIV (PWH) is roughly two-fold higher compared to HIV-negative persons [1]. Increased cardiovascular risk in PWH is multifactorial and includes chronic innate and adaptive immune activation, dyslipidaemias, insulin resistance, and other factors [2–4]. Prior studies have also implicated impaired vascular compliance, or a reduced ability for blood vessel walls to expand and contract passively with changes in pressure [5], as a common finding in PWH and a possible mechanism contributing to this increased CVD risk. [2, 6]. The decrease in vascular compliance has been attributed to heightened inflammation, immune activation, oxidative stress, and insulin resistance in PWH [7–11] and linked to reduced bioavailability of endothelial nitric oxide (NO) [12]. However, there are few studies of vascular compliance among PWH in sub-Saharan Africa (SSA), a region with the highest burden of HIV, an increasing prevalence of CVD among PWH, and a high proportion of low body mass index (BMI) individuals frequently underrepresented in prior studies.

Non-invasive methods for measuring vascular compliance include flow-mediated dilation, a measure of endothelial function and smooth muscle response to hypoxia, and pulse wave velocity (PWV), a measure of arterial distensibility or stiffness. Many studies have shown that aortic stiffness, measured as the velocity at which ventricular contraction of the heart, propagate along the arterial tree (distance/time [m/s]), as measured by carotid-femoral pulse wave velocity (cfPWV), a gold standard for aortic stiffness [13], is an independent risk factor for cardiovascular events (both fatal or non fatal) and all-cause mortality [14]. A systematic review and meta-analysis of 17 longitudinal studies in the general population found an increase of 1 m/s in PWV is associated with a 14% increase in CVD events and a 15% increase in mortality [14]. Furthermore, PWV measurement can be incorporated into routine clinical assessments and maybe useful for risk stratification [15–17]. In addition to cfPWV, the carotid-radial pulse wave velocity (crPWV) and pulse pressure are independently associated with cardiovascular events [18, 19]. These non-invasive measures of arterial stiffness are calculated from the duration and features of the arterial pulse propagated from the carotid to the femoral or radial arteries [20].

Additional parameters important to the assessment of vascular compliance include the central diastolic blood pressure (cDBP; the average minimal blood pressure value over one heartbeat), central pulse pressure (cPP; the maximal peak-to-peak amplitude of the pressure wave over one heartbeat), mean arterial pressure (MAP; the calculated average blood pressure value over one heartbeat), central systolic blood pressure (cSBP; the average blood pressure value during systole) and the augmentation pressure, or the increase in blood pressure induced by the early arrival of the reflected wave [21]. The augmentation index (AIX), the difference in the peak-to-peak amplitude of the pressure wave expressed as a percentage of pulse pressure, represents another important measure of vascular compliance [21, 22].

Impaired vascular function has emerged as an important contributor to CVD in studies of PWH from high resource countries with a relatively higher proportion of overweight and obese individuals[2, 6]. Most studies using non-invasive physiological measures of vascular function have reported impaired vascular compliance in overweight/obese PWH who are already at risk of CVD [10, 23]. Our group previously reported that lean PWH on ART for more than five years had increased arterial stiffness using the measure AIX [24]. However, reports of PWV measurements in PWH, especially lean individuals, have been inconsistent. While some studies show similar findings as in HIV-negative persons, others [25] show an increase in PWH [26]. Given the rising burden of CVD among individuals on long-term ART in SSA, where the majority of PWH reside [27], there is a critical need to understand cardiovascular risk factors specific to this region and population. Here, we assess whether underweight PWH on long term ART may have improved vascular compliance compared to normal weight and overweight individuals in Zambia, which could reflect a protective effect of low BMI for the development of CVD.

## Materials and methods

We conducted a cross-sectional study among PWH on long-term ART at the University Teaching Hospital (UTH) in Lusaka, Zambia between September 2018 and June 2019. The sample size of 91 was estimated using Epi info version, based on the 11% prevalence of HIV in Zambia and 80% power. This sample size was calculated prior to the time of data collection [28]. Simple random sampling was used to select participants. All PWH had been on a regimen of efavirenz, emtricitabine, and tenofovir disoproxil fumarate for more than five years. Persons who were pregnant or with any known CVD, rheumatologic disease, diabetes, or any other active infectious conditions aside from HIV, or with current tobacco use were excluded. Sociodemographic data such as history of tobacco use, alcohol intake, sex and age were collected using the WHO STEPS questionnaire [29]. Height, recorded in centimeters, and weight, in kilograms, was measured using the micro T3 PW-BMI digital physician scale. While taking the measurement, participants were requested to remove footwear or head gear, have feet together, arms on the side while facing ahead. BMI was categorized as < 18.5 kg/m^2^ (underweight), 18.5–24.9 kg/m^2^ (normal), and >  = 25.0 kg/m^2^ (overweight) [30].

### Arterial stiffness measurements

PWV was measured at the femoral and radial sites, in addition to AIX, pulse waveforms and central blood pressures, using the ALAM Complior Analyse device (ALAM medical, France). Non-invasive probes were applied to the skin’s surface over the carotid, femoral and radial arteries with participants lying in a supine position and after resting for a minimum of five minutes in a quiet room. Participants wore minimal clothing, jewellery was removed, and telephones and other personal electronics were placed in a separate room. Participants were not allowed to move, speak, or sleep during the measurements [20]. All measurements were performed by the same operator and reported as cfPWV, crPWV, and carotid femoral AIX (cfAIX) and carotid radial AIX (crAIX).

Aortic pressure waves can be classified into Types A, B, C and D according to the reflected wave’s timing and amplitude from peripheral sites [13]. Types A and B occur when the systolic peak (P2) occurs in late systole after a clear inflexion point. AIX is calculated as the difference between P2 and the pressure at the systolic shoulder (P1), expressed as a percentage of the pulse pressure. In Type A, the AIX is higher than 12 per cent, whereas it is between 0 and 12 per cent in Type B [13]. Type C waves have inflexion points after the systolic peak. In contrast, in Type D, no inflexion point is measured because the reflected wave arrives in early systole and merges with the incident wave [13]. Types A and B are characteristic of impaired vascular compliance. Type C is characteristic of typical vascular function, whereas Type D is commonly observed in age-related arterial changes in the elderly [13].

### Statistical analysis

Microsoft Excel (Microsoft, Redmond, Washington, USA) and STATA 14.2 (StataCorp LLC, College Station, Texas, USA) [31] were used for data analysis. To check the normality of the data, Q-Q plots were used and validated with the Shapiro–Wilk test. Homogeneity of variance was tested using Levene's test for one-way anova for endothelial function and BMI. The assumptions of normality and homoscedasticity of anova were not met, so the Kruskal Wallis test was used. Descriptive data were presented as medians and interquartile ranges for continuous variables and percentages for categorical variables. The relationships between various clinical and demographic factors across BMI categories were assessed using the Kruskal Wallis test. Univariate and multivariate linear regression were used to assess the relationships of endothelial function (crPWV, cfPWV, crAiX, and cfAiX) with sex, age and BMI category, as well as SBP, DBP, and MAP. *P* ≤ 0.05 was considered statistically significant [32].

## Results

Ninety one adult participants, comprised of 57 females and 34 males, were enrolled in the study; 38 participants (42%) were underweight, 43 (47%) normal weight and 10 (11%) overweight. The median ages were 41, 40 and 40 years, respectively (Table 1). Median central blood pressure measurements were also comparable across BMI categories and no significant differences were observed. A summary of the data is shown in Table 1.

**Table 1:** Clinical characteristics of study participants

Variables	Underweight (n = 38)	Normal weight (n = 43)	Overweight (n = 10)	*P*
Age, years*	41 (43–40)	40 (43–33)	40 (42–30)	0.56^ K^
Sex, Female	16 (42%)	27 (63%)	9 (90%)	0.01^C^
Alcohol use, yes	21 (55%)	22 (51%)	2 (20%)	0.13^C^
Tobacco use, yes	11 (29%)	4 (9%)	1 (10%)	0.06^C^
Height, cm	167.2 (174–158)	164.2 (169–159)	161.1 (168.4–156.5)	0.13^ K^
Waist hip ratio	0.81(0.41–1.14)	0.79 (0.56–0.97)	0.85 (0.75–1.02)	0.90^ K^
Central systolic blood pressure, mmHg	105 (114–101)	108 (120–100)	129 (137–121)	0.51^ K^
Central diastolic blood pressure, mmHg	76 (85–73)	77 (86–70)	84.5 (102–67)	0.47^ K^
Mean arterial pressure, mmHg	97 (100–89)	92 (104–81)	93 (100–89)	0.45^ K^
Carotid-radial augmentation index, crAIX	14.5 (39.7–8.9)	22.35 (42.5–11.35)	22.9 (38.3–6.3)	0.44^ K^
Carotid-femoral augmentation index, cfAIX	24.2 (33.98–5.9)	18.5 (33.6–8.9)	24.8 (42.1–13.2)	0.42^ K^
Carotid-femoral pulse wave velocity, m/s	7.9 (9.3–5.9)	6.9 (8.1–6.3)	7.0 (7.7–5.9)	0.36^ K^
Carotid-radial pulse wave velocity, m/s	10.0 (13.0–9.0)	10.0(11.2–8.4)	9.5 (11.6–8.0)	0.22^ K^
Pulse pressure, mmHg	37.5 (43–34)	37.8 (48.5–33.6)	41 (42–36)	0.51^ K^

^*^Medians and interquartile ranges and frequency. The Kruskal Wallis test used to compare continuous values across groups (K = kruskal walis), Chi square test used to compare categorical variables(C-Chi square). HIV, human immunodeficiency virus; PWH, persons with HIV; BMI, body mass index

Overweight participants had a 39% higher cfAIX compared to normal-weight participants after adjusting for age, sex and blood pressure and other factors (p > 0.02), while being underweight was associated with a 27% lower cfAIX (*P* = 0.01). None of the other demographic, anthropometry and hemodynamic parameters were significantly associated with cfPWV (Table 2).

**Table 2:** Regression analysis of carotid femoral: pulse wave velocity and augmentation index

	cfPWV		cfAIX	
	Regression coefficient (95% CI)	*P* value	Regression coefficient (95% CI)	*P* value
*Demography*				
Sex, Female	0.75 (− 3.50 to 5.00)	0.71	− 3.07 (− 23.3 to 17.2)	0.75
Age, years	0.06 (− 0.24 to 0.35)	0.70	0.69 (− 0.73 to 2.11)	0.32
Alcohol use	0.06 (− 2.45 to 2.56)	0.96	− 5.97 (− 17.9 to 5.94)	0.30
*Anthropometry*				
Normal weight	Ref			
Underweight	− 1.39 (− 5.11 to 2.33)	0.44	− 27.0 (− 44.7-(− 9.27))	**0.01**
Overweight	3.93 (− 2.57 to 10.4)	0.22	38.9 (7.88 to 69.8)	**0.02**
Waist-hip ratio	− 7.51 (− 19.6 to 4.57)	0.21	36.0 (− 21.5 to 93.5)	0.20
*Hemodynamic parameters*				
Central DBP, mmHg	0.08 (− 0.14 to 0.31)	0.43	− 0.03 (− 1.09 to 1.03)	0.95
Central SBP, mmHg	0.01 (− 0.15 to 0.16)	0.91	0.17 (− 0.57 to 0.91)	0.64
Pulse, beats/minute	0.07 (− 0.07 to 0.21)	0.32	− 0.58 (− 1.25 to 0.09)	0.09

cfPWV, carotid-femoral pulse wave velocity; cfAiX, carotid-femoral augmentation index. P values < 0.05 shown in bold

Table 3 shows the regression analysis of crPWV and crAIX. A unit increase in central diastolic blood pressure was associated with a 0.16 m/s increase in arterial stiffness as measured by crPWV (*P* = 0.03) when adjusted for other variables in the study. None of the other factors was significantly associated with crAIX.

**Table 3:** Regression analysis of carotid radial: pulse wave velocity and augmentation index

	crPWV		crAIX	
	Regression coefficient (95% CI)	*P* value	Regression coefficient (95% CI)	*P* value
*Demography*				
Sex, Female	0.98 (− 1.51 to 3.46)	0.41	11.4 (− 34.9 to 57.7)	0.61
Age, years	0.04 (− 0.13 to 0.21)	0.62	0.97 (− 2.23 to 4.17)	0.53
Alcohol use, Yes	− 1.32 (− 3.22 to 0.58)	0.16	1.88 (− 33.5 to 37.3)	0.91
*Anthropometry*				
Normal weight	Ref			
Underweight	1.89 (− 0.52 to 4.30)	0.11	− 28.6 (− 73.5 to 16.1)	0.19
Overweight	− 0.87 (− 5.05 to 3.32)	0.66	− 6.60 (− 84.5 to 71.2)	0.86
Waist-hip ratio	− 3.65 (− 11.4 to 4.05)	0.33	128 (− 14.9 to 272)	0.08
*Hemodynamic parameters*				
Central DBP, mmHg	0.16 (0.02 to 0.29)	**0.03**	− 0.28 (− 2.76 to 2.20)	0.81
Central SBP, mmHg	− 0.04 (− 0.14 to 0.06)	0.38	− 0.11 (− 1.95 to 1.73)	0.90
Heart rate, beats/minute	0.01 (− 0.06 to 0.09)	0.67	0.10 (− 1.24 to 1.43)	0.88

cfPWV, carotid-radial pulse wave velocity; cfAiX, carotid-radial augmentation index. P values < 0.05 shown in bold

### Waveform analysis

Table 4 shows the waveform distribution across BMI categories. The predominant waveform in all three BMI categories was type A, followed by type B. Overall, there was no significant difference in the waveforms between BMI categories (*P *value = 0.08).

**Table 4:** Waveform distribution across weight categories

BMI category	Waveform A	Waveform B	Waveform C	*P* value
Underweight (n = 38)	24 (63%)	13 (34%)	1 (3%)	0.08
Normal weight (n = 42)	26 (62%)	15 (36%)	1 (2%)	
Overweight (n = 9)	6 (67%)	1 (11%)	2 (22%)	

Waveform distribution across BMI categories compared by Chi square test. *P *value compares waveform distribution across the three BMI categories. Wave form data was unavailable for one normal weight and one overweight participant

## Discussion

In a cohort of PWH on a single ART regimen in Zambia, we found that PWH on long-term ART across BMI strata had similar measurements of both carotid-radial and carotid-femoral pulse wave velocities and augmentation indexes, suggesting a lower BMI may not offer substantial protection against impaired vascular compliance. Further, most participants had an AIX above 0%, which suggests that underweight PWH have a similar vascular phenotype as overweight PWH. A concordant relationship has been reported between augmentation index and pulse wave velocity in those at high risk of impaired vascular compliance [33, 34]. However, due to lack of a HIV negative control group, this could not be determined. We found that nearly all participants had either Type A or B waveforms, which represent the return of the reflective wave in systole instead of diastole [13]. Type C waveforms, considered desirable, (AIX < 0%) represent a return to the ascending aorta after ventricular ejection has ceased. However, typical in A and B waveforms, increases in the AIX cause decrease in the diastolic coronary pressure. These, typical in hypertensive patients, are characterised by increased ventricular afterload and increased myocardial workload and mass. Studies have also shown that the less compliant proximal arteries reduce the pressure gradient of proximal arteries to distal arteries. This may cause increased pressure in the microcirculatory system, a condition that could be harmful to areas of the brain and kidneys [35–37].

Vascular compliance measurements are modulated by vasomotor tone which is determined, in part, by endothelial function, the sympathetic nervous system and the renin angiotensin aldosterone system [38]. Our study reported no univariate differences in the haemodynamic parameters measured. Further, our result ranges are similar to other studies [39]. Further, increases in diastolic blood pressures were associated with increases in carotid radial pulse wave velocity. However, decreases in the bioavailability of nitric oxide can modulate vascular compliance independent of increases in the blood pressure [40, 41]. Thus, investigations are needed to determine the influence that sympathetic tone may have on aortic stiffness [38].

Long-term ART exposure has been associated with increased visceral fat and increasing CVD risk [42–44]. The association between ART and weight gain is yet to be determined but has been attributed to off target effects of the treatment [45]. The disposition of fat in central parts of the body increases one’s risk of cardiovascular disease [46]. Our study reported no significant differences in the waist hip ratio among groups. Further, using cfAIX, our results show that increased body mass is likely to increase the AIX and represent impaired vascular compliance while controlling for other variables. Indeed, obesity is a known risk factor for CVD, potentially mediated by endothelial dysfunction [47, 48]. Among the underweight, using cfAIX, our results show that an increase in body mass may decrease the AIX, restoring vascular compliance after controlling for other variables. This is similar to what Kumar and Samaras [49] described as healthy weight gain or a “return to health”. It however remains uncertain whether underweight PWH are protected against elevated arterial stiffness by their body composition.

Several studies have attributed impaired vascular compliance to ART. Nucleoside reverse transcriptase inhibitors such as abacavir have been implicated in causing endothelial dysfunction by decreasing the bioavailability of nitric oxide [50]. Studies have also shown that exposure of endothelial cells to protease inhibitors increases leukocyte recruitment and adhesion to these cells [51, 52]. This adhesion stimulates the increased secretion of cytokines such as interleukin 6 (IL6) and tumour necrosis factor (TNFα), which have been implicated in attenuating the production of nitric oxide [53]. Decreases in nitric oxide result in decreased vascular compliance. In contrast, Dorjee, Desai [54] reported that while some ART regimens such as abacavir and tenofovir based drug combinations have been associated with increased cardiovascular risk, tenofovir and emtricitabine-efavirenz were associated with a lower risk of acute myocardial infarction [54]. However, more studies are needed to validate these findings.

Our study had several strengths, but also some limitations. It is among the few describing arterial stiffness measures and waveforms to assess cardiovascular risk in PWH on ART, particularly among low BMI individuals underrepresented in prior analyses. Additionally, our study adds to the presently limited data on CVD risk factors among PWH in sub-Saharan Africa, the region with the highest burden of HIV. However, due to the cross-sectional design, causality could not be determined. Further, the study lacked an HIV negative control group and was conducted in a primarily urban population in Zambia. Further studies will be needed to assess the generalizability of the results to other populations in SSA as well as other regions.

## Conclusion

In SSA, underweight and overweight PWH had a similar distribution of vascular waveforms suggesting that low BMI may not confer substantial protection against impaired vascular compliance among individuals on long-term ART. As PWH can now survive decades on effective ART the burden of CVD continues to increase. Future studies of interventions to reduce CVD risk in PWH should include greater representation of low BMI individuals, as well as assessing effectiveness specifically in the context of the HIV epidemic in SSA.

## Data Availability

The data that support the findings of this study are available from the corresponding author, L.K., upon reasonable request.
